# Data, dialogue, and design: patient and public involvement and engagement for natural language processing with real-world cancer data

**DOI:** 10.3389/fdgth.2025.1560757

**Published:** 2025-05-15

**Authors:** Wuraola Oyewusi, Eliana M. Vasquez Osorio, Goran Nenadic, Issy MacGregor, Gareth Price

**Affiliations:** ^1^ Division of Cancer Sciences, School of Medical Sciences, The University of Manchester, Manchester, United Kingdom; ^2^ Radiotherapy Related Research, The Christie NHS Foundation Trust, Manchester, United Kingdom; ^3^ Department of Computer Science, The School of Engineering, The University of Manchester, Manchester, United Kingdom; ^4^ Vocal, NIHR Manchester Biomedical Research Unit, Manchester University NHS Foundation Trust, Manchester, United Kingdom

**Keywords:** patient and public involvement and engagement, PPIE, natural language processing, NLP, real world data, RWD, patient advocacy, ethical AI

## Abstract

**Introduction:**

This study describes the process and outcomes of a Patient and Public Involvement and Engagement (PPIE) event designed to incorporate patient perspectives into the application of Natural Language Processing (NLP) for analyzing unstructured free-text cancer medical notes. The analysis of routinely collected data aims to provide evidence to support clinical decision making in patient groups that are often under-represented in conventional clinical trials, highlighting the critical role of PPIE in responsibly implementing AI within healthcare. The study focuses on ensuring that NLP research reflects patient-centered and clinically relevant considerations.

**Methods:**

The event involved 13 participants: nine cancer survivors and caregivers, acting as contributors, and four researchers. These participants engaged in focus group discussions on three key topics: data use, consent preferences, and communication strategies for this type of research.

**Results:**

Some key findings included that two-thirds (6/9) of contributors preferred a national opt-out consent model for data use, while one-third (3/9) favored project-specific consent. They offered perspectives on data use, including how it is processed and stored. They also highlighted the importance of clear, accessible information about the research process to build trust and facilitate informed decision-making.

## 1 Introduction

In gathering evidence for cancer treatment, clinical trials are crucial. Clinical trials are the gold standard for evaluating treatment efficacy and safety (1), enabling rigorous testing and validation of medical interventions. However, to minimize bias and other factors, these trials are conducted within a controlled and narrow spectrum, which perpetuates issues like underrepresentation of patient populations, limiting generalizability (2). To improve this inclusivity and applicability, Real-World Data (RWD), which is data collected from routine patient care and other sources than traditional clinal trials (3), can enable learning from a broader patient range and provide insights into treatment effectiveness across diverse populations.

In recent years, the availability of RWD in healthcare has significantly increased due to the widespread adoption of digital health systems, particularly electronic health records (EHRs). Government policies, such as the US Health Information Technology for Economic and Clinical Health (HITECH) Act of 2009, have incentivized EHR implementation across hospitals and clinics, leading to improved data collection and access (4). In the United Kingdom, general practitioners (GPs) have been fully computerized for more than two decades, further illustrating the transition to digital health (5). As an example of digital data availability, clinical oncology practices now capture detailed information about patients' cancer care, including diagnosis, treatment, pathology, and radiology reports, which are often found in free text narrative and scanned documents (6). It is estimated that up to 80% of medical notes are unstructured, recorded in free text either typed or dictated by physicians (7). This leaves approximately 20% of RWD structured (e.g., in tables, coded data).

Structured data are more algorithm-friendly, easier to anonymize, and simpler to process. Converting unstructured data contained within free text into structured formats can expand the scope of useful data, reduce missingness and sparseness, thereby enhancing learning from all patients. In this context, Artificial Intelligence (AI) techniques, particularly Natural Language Processing (NLP) play a crucial role in transforming unstructured data into actionable insights.

NLP is the study of Natural Language rather than artificial language; it focuses on making sense of sequences like text and speech data. With NLP, computers can simulate understanding of text and spoken words in a way like humans by combining computational linguistics and rule-based modeling of human language with statistical, machine learning and deep learning models (8).

In this study, we explore patients' perspectives on the application of NLP to oncology medical notes. It was conducted as part of a broader research initiative applying NLP to real-world cancer data, with the goal of extracting structured insights from free-text clinical notes e.g., pathology reports, radiology reports to improve treatment evaluation and patient outcomes. The overarching aim of “real-world evidence” research is to make the evidence used to support clinical decision making more inclusive—currently many patient groups are under-represented in conventional clinical trial datasets, and as a result there is uncertainty around the best treatment strategies for many patients seen in cancer clinics. The unstructured nature of free text data presents unique challenges compared to structured data; even sophisticated anonymization methods may not fully guarantee the complete de-identification of personally identifiable information (PII). Residual identifiers within free text can still be present and may be cross-referenced with external sources.

This necessitates adopting more secure methodologies and ensuring our processes are both acceptable to and clearly communicated with patients and the public. Engaging with the focus community is important in addressing these questions, hence the need for Patient and Public Involvement and Engagement (PPIE).

Our key focus is to understand patients’ perspectives on the use of NLP in cancer free-text data, structured around three key themes: data use, research participation, and research communication. Within the data use theme, discussions explored how free-text medical notes are stored, processed, and safeguarded in NLP research. This included addressing concerns about anonymization techniques, secure data storage in approved repositories, and controlled access to ensure privacy protection. We also examined how patients prefer to consent to research involving the NLP processing of potentially identifiable written medical notes. Furthermore, we sought to identify the types of information that should be communicated about the project and the most effective methods for delivering this information. To explore these aspects, we conducted a PPIE event, fostering dialogue between patients, caregivers, and researchers. This collaborative approach not only enhances the ethical deployment of NLP in oncology but also ensures that the patients' voices are integral to the research process.

Patient and Public Involvement and Engagement (PPIE) refers to actively involving patients, carers, or other members of the community in health research design and implementation. These individuals are often end-users of healthcare solutions but traditionally lack a role in shaping the research. Their inclusion is essential, as they offer unique, lived experiences and perspectives that can enhance the research's relevance, ethical standards, and real-world applicability. Through PPIE, researchers work with ordinary people to shape and produce better research and result dissemination (9). Public involvement in research is defined as research being carried out “with” or “by” members of the public rather than “to”, “about” or “for” them. It is an active partnership between patients, carers, and members of the public with researchers that influences and shapes research (10).

In their work on building trust in AI for healthcare, Banerjee, et al. (11) advocated for the integration of patient and public perspectives, emphasizing that AI algorithms and work processes should be co-designed with patients and healthcare workers, specifically including patients with lived experience of the disease. To facilitate this, they propose the creation of a research advisory group (RAG) where patients are walked through the AI model building process, starting with simple models, to foster understanding and realistic expectations. This approach aims to counter the often-prevalent hype and negative narratives surrounding AI in healthcare, promoting adoption and acceptance by ensuring that patient perspectives, thoughts, and experiences are embedded into the research to improve its relevance and ethical grounding. Lammons et al. (12) also explored public and patient perspectives through a focus group study to understand perceptions of AI in healthcare. They identified key themes around the potential advantages of AI, including improvements in system efficiency, enhanced patient care, and better shared decision-making. However, they also highlighted concerns such as security, bias, access, public misunderstanding, and the loss of human touch in care. To address these challenges, Lammons et al. emphasized the importance of early and robust PPIE to not only safeguard patients but also to increase public acceptance and maximize the impact of AI on healthcare outcomes. Their findings underscore the need for incorporating diverse perspectives to ensure that AI technologies are both effective and aligned with patient values, ultimately fostering trust and ensuring a more patient-centered approach to AI implementation. In relation to public engagement specific to real-world free-text data, Ford et al. (13) conducted a citizens' jury study to explore public perspectives on sharing medical free-text data for research purposes. Over three days, 18 jurors deliberated on the ethical implications of using unstructured clinical information such as letters, reports, and notes often overlooked in research due to privacy concerns. While jurors generally supported sharing medical data for public health benefit, they were more cautious about free-text data. They expressed a preference for computer-assisted processing to extract information at scale, highlighting concerns about transparency in data use and privacy risks. The jurors recommended keeping patients informed about the use of their data and offering clear pathways for opting out of data sharing.

## 2 Materials and methods

**Figure 1:**
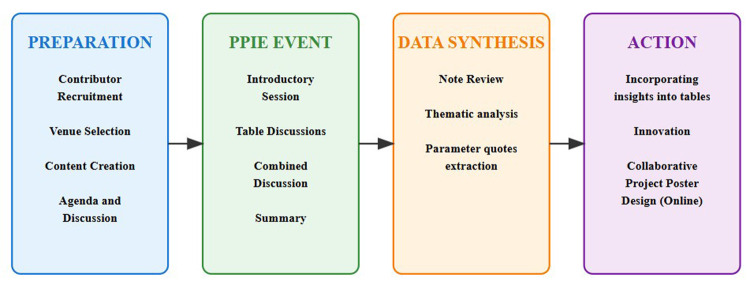
Methodology for a patient and public involvement and engagement for NLP on cancer medical notes.

This study employed a structured, participatory approach to deliver a PPIE event that engaged cancer survivors, caregivers, and researchers to explore patient perspectives on the application of Natural Language Processing (NLP) to cancer medical notes. The methodology is organized into four main phases: Preparation, PPIE Event, Data Synthesis and Action based on the study, as illustrated in Figure 1.

### 2.1 Preparation

The preparation phase focused on laying a solid foundation for the PPIE event by leveraging community networks and developing detailed event materials. The key activities include:

#### 2.1.1 Community engagement

We collaborated with Vocal, A local cancer-focused network focused on delivering services and innovative projects that bring patients, researchers, scientists and communities together to enhance health research.

#### 2.1.2 Recruitment of contributors

Contributors were recruited in collaboration with the PPIE specialist in the community. This ensured the inclusion of diverse voices, including cancer survivors and caregivers. The recruitment process emphasized diversity in lived experiences and demographic representation.

#### 2.1.3 Venue selection

A central and accessible location was selected to accommodate participants and promote inclusivity.

#### 2.1.4 Content and agenda development

Working alongside the PPIE specialist, the research team curated an agenda, prepared discussion materials, and crafted targeted questions to guide discussions during the PPIE event, the research team curated targeted questions addressing three central themes: Data Use, Research Participation, and Research Communication.

These themes were chosen to explore contributors' perspectives on how their medical data is utilized, the consent models preferred for such research, and the communication strategies that would effectively convey information about the project. Table 1 outlines the key questions and discussion points associated with each theme, providing a framework for participant engagement.

**Table 1:** Key questions asked during the PPIE event on NLP for written cancer medical notes.

Concept	Key questions	Discussion points/action
Data use	Contributors’ initial views on the use of written medical notes in cancer research	• What is important to patients and carers when working with potentially identifiable written medical note data? • What is important to consider for our research project?
Research participation	Contributors’ perspective on national data opt-out versus project specific opt-out for work with potentially identifiable written medical data	• Is the national data opt-out appropriate for our research project? Is a study specific opt-out also needed? • Vote: Is the national data opt-out or study specific opt-out most appropriate for our research project?
Research communication	Contributors’ opinions on information that needs to be disseminated about this work	• What type of information is important to communicate about our research project? • How should information be shared?

### 2.2 PPIE event

The PPIE event was a 2-h in-person focus group designed to foster meaningful discussions and collaborative learning among participants. The event included 13 participants: 9 contributors (cancer survivors and caregivers) and 4 facilitators (researchers). The key activities included:

Introductory Session: The event began with a presentation by a team member to establish context. This presentation outlined the specific objectives of our NLP research, which focuses on extracting structured data from oncology notes such as pathology reports, radiology notes and treatment summaries to analyze real-world cancer outcomes. It covered the limitations of clinical trials in cancer treatment, such as underrepresentation and high costs, using concrete examples to highlight how free-text clinical narratives capture valuable insights often missing from structured trial data. Additionally, the advantages of real-world data (RWD) for improving inclusivity and addressing disparities in care were discussed.

The presentation also addressed key risks and benefits associated with using medical notes in NLP research, demonstrating anonymization techniques with redacted cancer records to illustrate privacy preservation methods. The facilitator also addressed data identifiability, emphasizing that while NLP models and anonymization techniques such as redacting names and dates are designed to remove identifiable details, complete de-identification cannot always be guaranteed due to factors like rare diagnoses or distinctive writing styles. To mitigate these risks, data is stored in secure repositories, accessible only to vetted researchers under strict governance protocols. The discussion aimed to provide transparency about both the privacy safeguards in place and the limitations of anonymization, ensuring participants had a clear understanding of how their data would be used and protected. The facilitator also explained how participant feedback would directly shape ethical considerations, including consent models and patient communication strategies tailored to cancer data. Printed copies of the presentation were provided to each table for reference throughout the discussions.

#### 2.2.1 Table discussions

Two discussion tables were set up, each with 4–5 contributors and 2 facilitators. The contributors addressed the curated questions, documenting their insights on post-it notes affixed to large sheets for collective review. Facilitators took detailed notes to supplement participants' contributions.

#### 2.2.2 Combined discussion

After the table discussions, participants reconvened for a group session to consolidate findings, allowing for cross-group dialogue and consensus-building.

#### 2.2.3 Voting activity

To capture preferences on consent models, participants engaged in a voting exercise. They were asked to decide between the National Opt-Out and Study-Specific Opt-Out In the UK, National opt-out is patients' choice to block their health data from being used in non-essential research while Study specific opt-out is the choice to opt out of specific research project only (14). Results were tallied and formed part of the thematic analysis.

### 2.3 Data synthesis

Following the PPIE event, the research team employed a structured approach to analyze and summarize the collected data, using thematic analysis as the primary method. Thematic analysis is a method for identifying, analyzing, and reporting patterns and themes within qualitative data. It's used to find recurring ideas, concepts, or meanings within text, interviews, focus groups. The process was guided using the Braun and Clarke (15) thematic analysis framework.

#### 2.3.1 Familiarization

The research team immersed themselves in the data by reading and re-reading the notes, post-it contributions, and voting outcomes, ensuring a deep understanding of the content.

#### 2.3.2 Searching for themes

The team collated the initial codes into potential themes, considering broader patterns and the central research question. Key insights were categorized into themes corresponding to the event's focus areas (Data Use, Research Participation, and Communication). Commonalities, patterns, and divergent opinions were identified.

#### 2.3.3 Reviewing and naming themes

Themes were refined by checking them against the data to ensure that they accurately reflected the content and addressed the research question. Each theme was clearly defined, and concise names were generated to encapsulate the essence of each theme.

#### 2.3.4 Producing the PPIE results

The research team selected exemplar quotes that illustrated each theme, ensuring that the findings were both compelling and representative of the participant perspectives.

#### 2.3.5 Event report compilation

A detailed report summarizing the event's outcomes was compiled and shared with contributors via email, ensuring transparency and providing an opportunity for feedback.

### 2.4 Action

The PPIE event resulted in actionable insights that were integrated into the project's ethical framework and research methodology. Feedback from the event informed the refinement of the consent model and communication strategies. Contributors also collaborated on designing a project poster to disseminate findings, fostering shared ownership and co-production.

## 3 Results

The focus group discussions provided valuable insights into patient and public perspectives on the use of NLP in analyzing real-world cancer medical notes. Contributors raised key concerns regarding data usage, consent models, and research communication. We have synthesized the key questions about the concepts with exemplar quotes from notes related to each concept.

### 3.1 Data use theme

Understanding patient concerns and priorities about how their medical data is used is vital for designing ethical and patient-centered research. Participants expressed various viewpoints on topics like inclusivity, data accuracy, completeness, and anonymization.

Table 2 summarizes the questions raised and includes exemplar quotes that capture contributors' concerns and reflections, offering a nuanced perspective on their expectations and reservations regarding data use.

**Table 2:** Questions and exemplar quotes about data use from the contributors during the PPIE event on NLP for written cancer medical notes.

Data use theme	Exemplar quotes
Inclusion • How are you going to ensure inclusion of people from different ethnic backgrounds? Sometimes minority patients are excluded from medical research	“How are you going to ensure inclusivity, sometimes patients don't get a choice” “How will the research ensure inclusivity of people of protected character”
Data accuracy • How accurate is the data? Some contributors inferred that data accuracy is more important than privacy. • Lack of confidence about what is written, inferred and recorded in medical notes i.e., is the text data correct? • Concerns over bias in source data, e.g., when written medical notes don't give the full picture	“Quality of data entered” “My GP has very detailed notes on me electronically. I am not concerned at all” “The data is as good as the recorder” “Errors in letters are common e.g., typos” “Previous cancer history is important, would be missed?=Partial results/Accuracy” “Based on recorded data -> Questions not asked!” “DATA from handwritten notes accuracy, ‘does the person secretary have knowledge’”
Data completeness • How is data about patients treated at multiple centres included—would all the related and historical medical records be included in this work? • Why is the focus of the study on only cancer data and not others like mental health? • Will handwritten data be use in the study?	“Risk of incorrectly transcribing (Handwritten) Data ‘GARBAGE IN GARBAGE OUT!’” “Previous cancer history is important would be missed? = partial results/accuracy” “Summaries have a clinical edge rather than capturing the complete picture (e.g., reassurance vs. anxious).” “Is missed personal data screening a big risk because its partial and less specific/individual”
Data usage • Could the structured data be used to go back to patient to check accuracy of the data?	“If something historical is discovered that may affect patient prognosis, do you get in touch to address it? - I would hope so!” “HNA = Holisitc Needs Assessment. Background data/info should be captured by CNS”
Masking and anonymization • What is the balance between anonymization and missing important details in the dataset	“What if important information is masked”

### 3.2 Research participation theme

Consent is a cornerstone of ethical research, particularly when handling sensitive medical data. During the PPIE event, contributors were asked to discuss and vote on their preferred consent model, focusing on the comparison between the National Opt-Out and Project-Specific Opt-Out models. Table 3 presents the voting results and provides quotes from contributors that reflect their reasoning, highlighting the practical and ethical considerations that informed their preferences.

**Table 3:** Contributors’ opinion on research participation consent during the PPIE event on NLP for written cancer medical notes.

Research participation themes	Exemplar quotes
Voting result	One contributor mentioned that hospitals often do audits, and there is no extra need to ask for opt-out in those contexts
National opt-out: 6/9	
Project specific opt-out: 3/9	

### 3.3 Research communication theme

Effective communication is critical for ensuring patients understand the scope and implications of research involving their data. Participants discussed their expectations for clarity, accessibility, and inclusivity in research communication.

Table 4 provides a summary of the key themes and exemplary quotes from these discussions, illustrating the contributors' emphasis on simplicity, multilingual support, and diverse communication channels to reach all patient groups.

**Table 4:** Questions and exemplar quotes about research communication from contributors during the PPIE event on NLP for written cancer medical notes.

Research communication themes	Exemplar quotes
Consent • Does this study include international patients? • Will opt-outing out of the research affect a person's treatment progress and standard of care?	“Just say ‘research to improve future patient care’ nothing more complex”
Inclusion • Is there provision for non-English speakers? • Digital based solution excludes some patients • Why is the study covering only a few years as treatments can change—will data be out of date? • Why is this study only at the Christie?	“How will the research ensure inclusivity of people protected characteristics” “2020–2014 Old DATA not Current”
Data privacy and security • What is the provision against data leaks in this project? • What is the procedure to mitigate date exposure? • Commercial use concerns	“Is missed personal data screening a big risk because its partial and less specific/individual i.e., don't worry”
Research info dissemination • Leaflets • Support groups • QR codes • Online website • On screen on TVs • Multilingual	“Comm. - Information on what the research is about • What is being collected • How it is going to be used • What will the outcome be or goal • Who will be involved. Cohort of patients? - what to do if you want to be involved?/How not to be involved? - who to ask if you have QS”

## 4 Discussion

Contributors emphasized the need for clarity and simplicity in how research is communicated to patients. The emphasis on accessibility especially for non-English speakers and those with limited digital literacy was particularly significant. As one participant succinctly put it: “Just say ‘research to improve future patient care’ nothing more complex.” This feedback highlights the importance of ensuring that research communication is straightforward and inclusive, particularly for vulnerable patient groups who may face barriers to understanding complex medical and research terminology.

Our study underscores the importance of incorporating patient and public perspectives in the design of cancer research, particularly in the context of emerging technologies such as Natural Language Processing (NLP) and other AI techniques. The insights gained are also relevant to other healthcare applications. While much of the literature on NLP in healthcare emphasizes algorithmic performance, there is a significant gap in understanding the implications for patients whose data is used. This study addresses that gap by documenting the process and findings of a PPIE event focused on NLP applications in cancer medical notes.

Contributors offered nuanced perspectives on data privacy, revealing that privacy preferences are not as rigid as traditionally assumed. Many were comfortable with reduced anonymization when they trusted the researchers and the research purpose. This challenges the conventional belief that stringent anonymization is universally prioritized and highlights the importance of a balanced approach. Initially, to enhance patient record privacy, we considered using data at least 2 years old, aiming to minimize the risk of identifying patients still actively seen in clinics. However, the participants thought that using such a time frame was exclusionary and may bias analyses, and, as for degree of anonymization, were comfortable with any risk provided they thought the research important. In response we expanded our inclusion window for using cancer medical notes. This decision, informed by the PPIE group, directly impacted our data use timelines, demonstrating how patient perspectives can influence real-world clinical practice.

Based on the voting results on the preferred consent model, the majority favored the National Opt-Out system, which would allow patients to opt out of research unless they explicitly choose to participate. This approach was seen as more practical and less intrusive than requiring active consent for each individual study and notably aligns with the UK's existing NHS Digital opt-out framework (14). However, the significant minority preference for Project-Specific Opt-Out highlights the diversity of opinion, suggesting policymakers should consider hybrid models in similar studies that maintain national-level efficiency while enabling granular control for sensitive studies. This balance could address ethical concerns while facilitating large-scale NLP research.

Communication emerged as a key concern, with contributors emphasizing the need for clarity and simplicity. The emphasis on accessibility—particularly for non-English speakers and those with limited digital literacy—was especially significant. As one participant succinctly put it: “Just say ‘research to improve future patient care’ nothing more complex.” This feedback underscores how standardized, plain-language communication guidelines could bridge gaps in understanding, particularly for vulnerable groups. Together, these findings demonstrate how patient-centered governance of medical NLP must address both consent flexibility and information accessibility to maintain public trust.

While our findings provide valuable insights, there are several limitations to consider. One key limitation is the recruitment of a small and geographically localized sample of participants. Only nine participants were included. Recruitment relied on voluntary participation, which may have self-selected individuals with stronger opinions on data usage. Although efforts were made to include individuals with diverse backgrounds, selection bias may have influenced the perspectives captured, this may not fully represent the views of the broader population. Future research could benefit from engaging a more diverse and larger sample to ensure that the findings are generalizable.

Additionally, while the focus group format was effective in fostering in-depth discussions, it may not have captured the full spectrum of participant perspectives. The group setting may have introduced social desirability bias, where participants expressed views, they believed to be more acceptable to researchers or peers.

Future studies could employ a combination of methods, such as surveys and individual interviews, to ensure broader representation and to gather both qualitative and quantitative insights. Moreover, our analysis relied on real-time facilitator notes and participant contributions on post-it notes. The inclusion of audio recordings and verbatim transcriptions in future research could enrich data collection, enabling a more comprehensive thematic analysis.

Our study also contributes to the growing body of literature on PPIE in healthcare research. Similar studies have explored public involvement in areas such as vitamin deficiencies (16), adverse event reporting (17), optimal Vitamin D status (18) and long COVID (19). However, to the best of our knowledge, our study is the first to document the PPIE process specifically for NLP applied to cancer medical notes. By addressing this underexplored intersection of AI and sensitive health data, our findings provide a foundation for developing socially and ethically grounded healthcare technologies.

## 5 Conclusion

In our work, we highlight the transformative role of Patient and Public Involvement and Engagement (PPIE) in designing ethically sound and patient-centered healthcare research, particularly in the application of Natural Language Processing (NLP) to cancer medical notes. The PPIE was centered around three core themes related to data use, research participation, and research communication.

Several insights were derived from the contributors, directly shaping our research approach. Regarding data use, a need to balance data privacy with data utility was evident, with many participants expressing a willingness to accept less stringent anonymization when trust was established. Regarding research participation, the majority (66.6%) favored the National Opt-Out consent model, while all contributors stressed the importance of clear, inclusive, and accessible communication strategies.

These findings, rooted in patient lived experience, have fundamentally refined our research design, demonstrating the invaluable impact of PPIE. As this is part of a broader project on applying NLP to cancer free-text medical notes, adjustments include broadening the data use timeline to incorporate a wider range of patient data, aligning with the majority preference for the National Opt-Out model, and developing communication strategies that are inclusive and comprehensible to diverse patient groups. Moving forward, we will continue to prioritize participant feedback to ensure that our research remains ethical, inclusive, and responsive to patient concerns. This work represents a significant step toward responsibly integrating AI into healthcare, ensuring that patient lived experience and preferences are central to the research process.

## Data Availability

The original contributions presented in the study are included in the article/Supplementary Material, further inquiries can be directed to the corresponding authors.
